# Research progress on intestinal probiotics-mediated protection of the intestinal mucosal barrier in ulcerative colitis

**DOI:** 10.3389/fimmu.2026.1943748

**Published:** 2026-09-28

**Authors:** Guiping Ma, Shurong Deng

**Affiliations:** 1 Central Laboratory, Beijing University of Chinese Medicine Affiliated Shenzhen Hospital (Longgang), Shenzhen, China; 2 Department of Pharmacy, The First Affiliated Hospital of Jinan University, Guangzhou, China

**Keywords:** gut microbial community, intestinal epithelial barrier, mucosal immune regulation, probiotic interventions, short-chain fatty acid metabolites, ulcerative colitis

## Abstract

Ulcerative colitis (UC) is a major subtype of inflammatory bowel disease characterized by a chronic, relapsing course. Its pathogenesis involves complex interactions among gut microbial dysbiosis, epithelial barrier disruption, dysregulated immune responses, and oxidative stress. Impairment of the intestinal mucosal barrier facilitates the translocation of luminal antigens, bacteria, and microbial metabolites into the mucosa, thereby aggravating local inflammation. Probiotics may help maintain barrier homeostasis by reshaping the gut microbial community, reinforcing epithelial and mucus barriers, modulating mucosal immunity, and reducing oxidative injury. This review summarizes recent evidence on the protective effects of probiotics, including *Lactobacillus* spp., *Bifidobacterium* spp., and *Akkermansia muciniphila*, on the intestinal mucosal barrier in UC. Current findings suggest that probiotics can inhibit pathogen adhesion and colonization, enhance the production of beneficial metabolites such as short-chain fatty acids and tryptophan-derived compounds, and promote the expression of tight-junction proteins, including ZO-1, occludin, claudin-1, and claudin-4, as well as mucins such as MUC1 and MUC2. These effects may be mediated through the regulation of NF-κB, NLRP3, aryl hydrocarbon receptor (AhR), mitogen-activated protein kinase (MAPK), and related signaling pathways, thereby attenuating inflammation and oxidative stress and promoting epithelial repair. Combination strategies involving probiotics and prebiotics, traditional Chinese medicine-derived components, postbiotics, fecal microbiota transplantation, or novel delivery systems may improve microbial engraftment, enhance colon-targeted delivery, and strengthen microbiota remodeling and barrier repair. Nevertheless, the available evidence remains limited by substantial strain specificity, insufficient clinical validation, and the absence of standardized intervention protocols. Further multi-omics studies and well-designed clinical trials are warranted to clarify the efficacy, mechanisms, optimal dosing, and safety of these interventions in UC.

## 1 Introduction

Ulcerative colitis (UC) is a major subtype of inflammatory bowel disease (IBD), characterized by chronic, relapsing inflammation that primarily affects the colonic and rectal mucosa and, in some cases, extends into the submucosa (1). Its common clinical manifestations include abdominal pain, diarrhea, mucopurulent bloody stools, tenesmus, and recurrent disease flares (2). UC is typically associated with a prolonged and relapsing disease course. Patients with severe or refractory disease may develop complications such as massive gastrointestinal bleeding, toxic megacolon, intestinal perforation, and an increased long-term risk of colorectal cancer, all of which can substantially impair quality of life (3). Current therapeutic options include aminosalicylates, corticosteroids, immunosuppressants, biologics, and small-molecule targeted agents (4). Although these therapies can induce and maintain clinical remission in many patients, their effectiveness is limited by interindividual variability in treatment response, disease recurrence, adverse effects associated with long-term therapy, and considerable economic burden (5–7). Therefore, adjunctive strategies that target key pathological processes and improve long-term disease management remain necessary.

Disruption of the intestinal mucosal barrier and gut microbial dysbiosis are central events in the initiation and progression of UC (8, 9). Under physiological conditions, the gut microbiota forms a complex ecological community that contributes to nutrient metabolism, short-chain fatty acid (SCFA) production, immune maturation, epithelial integrity, and colonization resistance against pathogens (10). In UC, microbial dysbiosis is commonly characterized by reduced microbial diversity, depletion of beneficial commensal bacteria, expansion of opportunistic pathogens, and impaired microbial metabolic function (11, 12). These alterations may weaken colonization resistance, compromise epithelial tight junctions and mucus-layer integrity, increase intestinal permeability, and facilitate the translocation of luminal bacteria, antigens, and microbial metabolites into the mucosa (13, 14). The resulting activation of innate and adaptive immune responses further aggravates inflammation and epithelial injury, thereby establishing a self-perpetuating cycle of “microbiota dysbiosis-barrier disruption-mucosal inflammation”.

Accordingly, targeted restoration of beneficial microbial communities and their metabolic functions has emerged as a potential approach to alleviating mucosal barrier injury in UC. Probiotics are defined as live microorganisms that confer health benefits to the host when administered in adequate amounts (15). Several probiotics, including *Lactobacillus* spp (15)., *Bifidobacterium* spp (16)., *Akkermansia muciniphila* (17), and certain *Parabacteroides* species (18), have demonstrated the potential to regulate intestinal microecology, reinforce mucosal barrier function, and modulate host immune responses in experimental UC models. Their proposed mechanisms include competition with pathogenic microorganisms for nutrients and adhesion sites, production of antimicrobial substances, enhancement of epithelial tight-junction protein expression, promotion of mucus and mucin secretion, and modulation of microbial metabolites, particularly SCFAs and tryptophan-derived metabolites (19–21). In addition, probiotics and their bioactive products may regulate inflammatory and barrier-related signaling pathways, including Toll-like receptor (TLR), nuclear factor-kappa B (NF-kB) (22), aryl hydrocarbon receptor (AhR), NLR family pyrin domain-containing 3 (NLRP3), and AKT/mTOR/HIF-1alpha signaling pathways (22–25).

Nevertheless, the therapeutic effects of probiotics in UC are not universal and should not be generalized across microbial genera or species. Their efficacy may vary substantially according to strain-specific characteristics, dose, formulation quality, viability, treatment duration, baseline gut microbiota composition, dietary pattern, disease activity, mucosal barrier status, and concomitant medications. Importantly, current evidence remains predominantly derived from *in vitro* and animal studies, whereas high-quality human clinical evidence is comparatively limited and heterogeneous. Moreover, in patients with severe active UC, profound mucosal barrier disruption, immunocompromised status, indwelling vascular catheters, or a high risk of bacteremia, the safety of live probiotic administration should be carefully assessed. Therefore, probiotics should be regarded as potential adjunctive interventions rather than substitutes for established standard-of-care therapies.

Previous reviews have primarily focused on the general anti-inflammatory effects of probiotics or their broad roles in regulating gut microbiota in UC. In contrast, the present review adopts intestinal mucosal barrier protection as its central framework and systematically examines the roles of probiotics in microbiota remodeling, tight-junction restoration, mucus-barrier repair, SCFA- and tryptophan-derived metabolite signaling, immune homeostasis, and oxidative stress regulation. We further summarize emerging microbiota-based strategies, including postbiotics, probiotic-derived extracellular vesicles, synbiotics, probiotic-traditional Chinese medicine combinations, sequential fecal microbiota transplantation (FMT)-probiotic interventions, and colon-targeted delivery systems. By integrating mechanistic evidence with translational challenges, this review aims to provide an updated perspective on the potential of probiotic-based interventions to protect the intestinal mucosal barrier and support UC management.

## 2 UC and intestinal mucosal barrier injury

### 2.1 Composition and function of the intestinal mucosal barrier

The intestinal mucosal barrier is the first line of defense against the entry of pathogenic microorganisms, toxins, and food antigens into the internal environment (26). It is mainly composed of mechanical, chemical, immune, and microbial barriers (27). The mechanical barrier is formed by intestinal epithelial cells and intercellular junctional complexes (27). Among these, tight junction proteins, such as zonula occludens-1 (ZO-1), Occludin, and Claudins, are essential for maintaining epithelial integrity and selective permeability (28).

The chemical barrier mainly consists of the mucus layer secreted by goblet cells (29), mucins (30), antimicrobial peptides (31), lysozyme (32), and bile acids (33), which prevent direct contact between pathogenic microorganisms and epithelial cells. The immune barrier is composed of gut-associated lymphoid tissue, immune cells in the lamina propria, secretory immunoglobulin A (SIgA) (34), and various cytokines, and maintains a dynamic balance between immune tolerance and anti-infective defense. The microbial barrier consists of commensal bacteria colonizing the intestinal lumen and mucus layer; these bacteria suppress pathogen colonization by competing for nutrients and adhesion sites, producing antimicrobial substances, and regulating host immune responses.

### 2.2 Abnormalities of the intestinal mucosal barrier in UC

The intestinal mucosa of patients with UC commonly shows epithelial cell injury, reduced goblet cell numbers, thinning of the mucus layer, decreased expression of tight junction proteins, and increased intestinal permeability (35–37). Once barrier integrity is compromised, luminal bacteria, lipopolysaccharide, and other antigenic substances can more readily penetrate the mucosa and enter the lamina propria (36, 38). This process activates macrophages, dendritic cells, neutrophils, T cells, and other immune cells, inducing the release of pro-inflammatory cytokines such as tumor necrosis factor-alpha (TNF-α), interleukin-1 beta (IL-1β), IL-6, and IL-17, which further aggravate epithelial injury and inflammation (36).

Abnormal tight junction structure is an important molecular basis of intestinal barrier dysfunction in UC (39). Reduced expression or disordered distribution of ZO-1, Occludin, and Claudins can increase paracellular permeability, thereby promoting bacterial translocation and persistent inflammation (40, 41). Injury to the mucus barrier is also closely associated with UC. Muc2 is the predominant secreted mucin in the colonic mucus layer (42), and its reduction weakens the spatial separation between luminal bacteria and the mucosal surface, allowing the intestinal microbiota to come into direct contact with epithelial cells and trigger inflammatory responses (43). Therefore, restoration of the intestinal mucosal barrier is not only an important step in alleviating inflammation in UC, but also a potential strategy for preventing disease relapse.

## 3 Gut microbiota dysbiosis in UC

The gut microbiota plays a central role in maintaining host nutrient metabolism, mucosal immunity, and barrier homeostasis (44, 45). Patients with UC commonly exhibit structural and functional abnormalities of the gut microbiota, characterized mainly by reduced alpha diversity, depletion of beneficial commensals, expansion of opportunistic pathogens, and altered microbial metabolic functions (46, 47). Decreased abundance of beneficial bacteria such as *Bifidobacterium* (48), *Lactobacillus* (49), and *Akkermansia muciniphila* (50) may reduce short-chain fatty acid production, impair mucosal barrier repair, and weaken immune tolerance. In contrast, enrichment of pro-inflammatory bacteria such as members of the *Enterobacteriaceae* may aggravate intestinal inflammation by producing endotoxins, enhancing oxidative stress, and activating inflammatory pathways (51, 52).

Microbiota dysbiosis can contribute to the course of UC through several mechanisms. On the one hand, the depletion of beneficial bacteria weakens colonization resistance against pathogens, making pathogenic bacteria more likely to adhere to the intestinal epithelium and invade the mucosa (53). On the other hand, changes in microbial metabolites can affect epithelial energy supply and immune cell differentiation (54). For example, butyrate is an important energy source for colonocytes and is also involved in maintaining regulatory T-cell differentiation and suppressing inflammatory responses (55). A reduction in butyrate-producing bacteria in UC may lead to impaired epithelial energy metabolism and disruption of immune homeostasis (56). In addition, abnormalities in tryptophan, bile acid, and lipid metabolism are closely related to inflammatory responses and barrier dysfunction in UC (57).

Accordingly, targeted modulation of the gut microbiota and restoration of beneficial bacteria and their metabolic functions represent important approaches to improving intestinal mucosal barrier injury in UC. As interventions that can directly or indirectly regulate the intestinal microecology, probiotics have shown promising potential in the prevention and treatment of UC (**Figure 1**).

**Figure 1:**
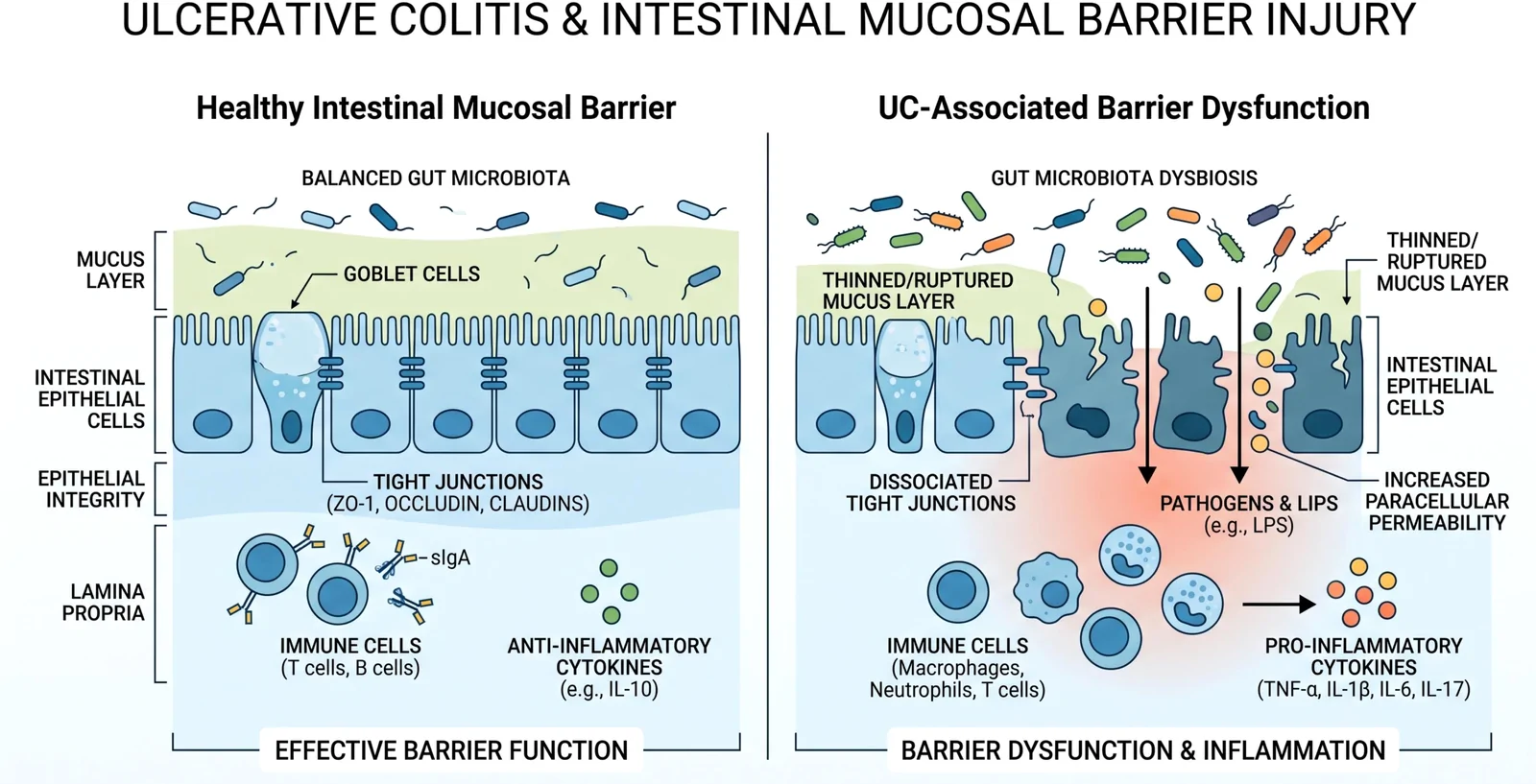
Alterations in the intestinal mucosal barrier under healthy and ulcerative colitis-associated conditions. Schematic representation of the structural and immunological differences between a healthy intestinal mucosal barrier and ulcerative colitis (UC)-associated barrier dysfunction. Under physiological conditions, a balanced gut microbiota, intact mucus layer, functional goblet cells, well-organized intestinal epithelial cells, tight junction proteins including ZO-1, Occludin, and Claudins, secretory IgA, and anti-inflammatory cytokines such as IL-10 collectively maintain epithelial integrity and mucosal homeostasis. In UC, gut microbiota dysbiosis, thinning or rupture of the mucus layer, goblet cell impairment, disruption of tight junctions, and increased paracellular permeability promote the translocation of pathogens and lipopolysaccharide (LPS) into the lamina propria. These changes activate immune cells, including macrophages, neutrophils, and T cells, and induce the release of pro-inflammatory cytokines such as TNF-α, IL-1β, IL-6, and IL-17, thereby contributing to sustained intestinal inflammation and barrier injury.

## 4 Mechanisms by which probiotics protect the intestinal mucosal barrier

### 4.1 Regulating gut microbiota structure and enhancing colonization resistance

Gut microbiota dysbiosis is an important microecological basis for the occurrence and development of UC, and is mainly characterized by reduced microbial diversity, depletion of beneficial commensal bacteria, and expansion of opportunistic pathogens (58). As an important intervention strategy for regulating intestinal microecology, probiotics can enhance the intestinal microbial barrier by reshaping microbial composition, restoring dominant beneficial bacterial populations, and inhibiting the colonization of potential pathogens, thereby contributing to the protection of the intestinal mucosal barrier (59). The underlying mechanisms mainly include competition for nutrients and adhesion sites, production of lactic acid, acetic acid, bacteriocins, and other antimicrobial substances, and reduction of the local pH in the intestinal lumen, thereby suppressing the adhesion, invasion, and excessive proliferation of pathogenic or opportunistic bacteria (60). In addition, some probiotics can establish relatively stable interactions with intestinal epithelial cells and the mucus layer (61), participate in the maintenance of intestinal biofilm-like structures (62), and reduce pathogen adhesion to epithelial cells through spatial occupation and niche competition, thereby preserving intestinal epithelial integrity and enhancing colonization resistance (63).

Under pathological conditions of UC, inflammatory responses and barrier disruption can alter the redox environment of the intestinal lumen and the availability of nutritional substrates, conferring a growth advantage on facultative anaerobes and certain opportunistic pathogens (64). For example, increased levels of oxygen and nitrate in the inflamed intestine can promote the expansion of facultative anaerobes such as *Escherichia coli*, which may further aggravate mucosal injury through lipopolysaccharide and other pro-inflammatory factors (65). Therefore, probiotic-mediated regulation of microbial community structure is not simply a matter of “increasing beneficial bacteria” or “reducing harmful bacteria”; rather, it involves restoring the dominance of anaerobic commensals, improving the intestinal luminal microenvironment, and limiting niche occupation by opportunistic pathogens, thereby establishing a more stable microecological network (66). Relevant studies have shown that modulation of the gut microbiota can significantly restore α-diversity in dextran sulfate sodium (DSS)-induced colitis mice, improve shifts in β-diversity, and reduce the relative abundance of *Pseudomonadota* and opportunistic pathogens such as *Escherichia*, *Proteus*, and *Morganella*, while increasing the abundance of beneficial bacteria such as *Lactobacillus* and *Clostridia*-related taxa (67, 68). These findings suggest that normalization of microbial community structure is closely associated with the alleviation of mucosal inflammation.

With respect to probiotic intervention, *Bifidobacterium animalis subsp*. BLa80 can alleviate UC-related pathological alterations by reshaping the gut microbiota (69) (**Table 1**). *Bifidobacterium infantis* FJSYZ1M3 can improve gut microbiota dysbiosis in UC mice, increase microbial species richness, reduce the relative abundance of certain harmful bacterial genera, and increase butyrate levels in cecal contents (70). As an important short-chain fatty acid, butyrate not only provides energy for colonic epithelial cells but also promotes mucus secretion, enhances the expression of tight junction proteins (71), and suppresses inflammatory responses (72). Therefore, probiotic-mediated restoration of microbial community structure and improvement of metabolic function have synergistic significance. Probiotics can not only directly restrict pathogen expansion through colonization resistance but also indirectly promote intestinal mucosal barrier repair by increasing beneficial metabolites such as short-chain fatty acids and butyrate (73, 74).

**Table 1:** Representative probiotics/postbiotics for intestinal mucosal barrier protection in UC.

Representative probiotic/derivative	Study model	Major therapeutic indicators	Proposed mechanisms
*Bifidobacterium animalis* subsp. *lactis* BLa80	UC-related experimental model	Improved colonic pathology and microbial composition	Reshapes gut microbiota and supports restoration of microbial homeostasis
*Bifidobacterium infantis* FJSYZ1M3	DSS-induced colitis mice	Increased microbial richness and cecal butyrate; reduced harmful taxa	Restores microbiota composition; promotes SCFA production and barrier repair
*Lactobacillus reuteri* ZY15	Experimental intestinal inflammation model	Increased Muc1, Muc2, ZO-1, Occludin, and Claudin-1; reduced IL-1beta and TNF-alpha	Enhances tight junctions and mucus barrier; suppresses inflammatory responses
*Limosilactobacillus reuteri* DSM 17938-derived postbiotics/EVs	5-fluorouracil-injured Caco-2 and primary intestinal epithelial cells	Improved epithelial barrier recovery and junction-related function	Cell-free supernatant, exopolysaccharides, and EVs support epithelial repair
*Akkermansia muciniphila*	DSS-induced colitis mice	Reduced inflammatory cytokines; improved mucosal integrity	Regulates mucus-associated ecology, tryptophan metabolism, and AhR-related signaling
*Bifidobacterium longum* Bif10/Bif11 plus maltose	Experimental colitis model	Increased butyrate, IL-10, IL-22, RegIIIbeta, and S100A8	Enhances SCFA-mediated immune regulation, antimicrobial defense, and mucosal protection
*Parabacteroides distasonis*	Experimental UC-related models	Reduced inflammation and improved barrier-associated outcomes	Regulates Th17/Treg balance, microbial ecology, and epithelial barrier repair
Multi-strain probiotic formulations	Animal models and clinical studies	Variable effects on disease activity, inflammation, microbiota, and barrier markers	Potential complementary actions across microbial regulation, metabolism, junction repair, and immune modulation; synergy requires validation

The proposed mechanisms listed in the table are derived primarily from preclinical studies and should not be interpreted as evidence of established clinical efficacy.

Notably, some studies further suggest the existence of a “microbiota-metabolite-host” cascade among microbial regulation, host inflammatory pathways, and barrier function (75). For example, in DSS-induced colitis models, intervention enriches beneficial bacteria such as *Ligilactobacillus animalis*, which is associated with increased levels of microbial metabolites such as 2,3-dihydroxybenzoic acid (76). These metabolites can upregulate the expression of tight junction-related molecules, including ZO-1, Occludin, and Claudin, and inhibit IL-6/SOCS3-related inflammatory signaling, thereby promoting intestinal mucosal barrier repair (76). Meanwhile, the abundance of beneficial bacteria is negatively correlated with the disease activity index, histopathological scores, and inflammatory factor levels, but positively correlated with MUC2 expression, colon length, and mucus layer integrity (77). These observations further indicate that restoring beneficial bacterial populations and suppressing the expansion of opportunistic pathogens are important mechanisms for improving intestinal mucosal injury in UC.

In summary, probiotics can effectively correct UC-associated intestinal microecological dysbiosis by regulating gut microbiota structure, enhancing colonization resistance, and improving microbial metabolic function. Their core effects are not limited to direct antagonism against pathogens; rather, they provide multilayered protection for the intestinal mucosal barrier by promoting the restoration of beneficial commensals, suppressing the expansion of opportunistic pathogens, increasing the levels of protective metabolites such as short-chain fatty acids, and improving the intestinal luminal ecological environment.

### 4.2 Strengthening tight junctions and reducing intestinal permeability

Tight junction proteins in the intestinal epithelium are key molecules for maintaining the integrity of the mechanical barrier. As described above, probiotics upregulate the expression of tight junction proteins, including ZO-1, Occludin, Claudin-1, and Claudin-4, thereby strengthening intercellular connections, reducing intestinal permeability, and limiting the translocation of pathogens and toxins.

*Lactobacillus reuteri* ZY15 has been reported to enhance intestinal barrier function by upregulating the mRNA and protein expression of Muc1, Muc2, Occludin, ZO-1, and Claudin-1, while reducing levels of pro-inflammatory cytokines such as IL-1β and TNF-α, thereby attenuating intestinal inflammation (25). Mohsen et al. showed that intervention with *Lactobacillus reuteri* significantly alleviated weight loss and colon shortening in mice with DSS -associated intestinal mucosal injury, reduced histopathological damage, and upregulated the expression of ZO-1, Occludin, Claudin-4, Muc1, and Muc2 (78). *Limosilactobacillus reuteri* DSM 17938, specifically cell-free supernatant, exopolysaccharides, and extracellular membrane vesicles, can support epithelial barrier recovery following 5 fluorouracil-induced injury (79) (**Table 1**). Although this study focused on a chemotherapy-induced barrier injury in both enterocyte-like Caco-2 cells and primary human intestinal epithelial cells, its findings on probiotic-mediated repair of tight junctions and the mucus barrier provide useful evidence for understanding mucosal barrier protection in UC model.

### 4.3 Promoting mucus secretion and mucin expression

The mucus layer is an important defensive structure on the intestinal epithelial surface, preventing bacteria, toxins, and antigens from directly contacting epithelial cells. Patients with UC often show reduced goblet cell numbers, insufficient mucus secretion, and decreased Muc2 expression, resulting in weakened mucosal protection (80, 81). Probiotics can promote goblet cell function, increase mucus secretion, and enhance the gene and protein expression of mucins such as Muc1 and Muc2, thereby strengthening the chemical barrier.

Probiotics such as *Lactobacillus* can improve the local intestinal microenvironment by promoting mucus secretion and mucin expression, preventing pathogen adhesion, and facilitating epithelial repair (82). *Akkermansia muciniphila*, a typical mucus-associated commensal bacterium, participates in mucus layer metabolism and regulation of barrier function. Studies have shown that the abundance of *A. muciniphila* is usually reduced in fecal or intestinal samples from patients with UC (83). In DSS-induced mouse models of UC, oral administration of *A. muciniphila* alleviates mucosal inflammation, preserves intestinal barrier integrity, and reduces inflammatory cytokine levels.

### 4.4 Regulating short-chain fatty acids and other microbial metabolites

One important mechanism by which probiotics protect the intestinal mucosal barrier in UC is the remodeling of the intestinal metabolic network, particularly the regulation of microbial signaling molecules such as SCFAs, tryptophan metabolites, bile acid metabolites, and lipid metabolites. SCFAs, mainly including acetate, propionate, and butyrate, are primarily produced by microbial fermentation of dietary fiber and polysaccharides. Among them, butyrate is not only an important energy source for colonocytes (83), but also regulates inflammatory responses, regulatory T-cell differentiation, and epithelial repair through G protein-coupled receptors and histone deacetylase inhibition (84, 85).

Evidence indicates that *Bifidobacterium* can produce SCFAs and activate SCFA/ERK/MAPK/MSK signaling pathways, thereby upregulating tight junction proteins such as ZO-1, Occludin, and Claudin-1, as well as mucins such as MUC2 and MUC3 (86, 87). This promotes the localization of junctional complexes to the cell membrane and strengthens both the mechanical and mucus barriers of the intestinal mucosa. *B. infantis* FJSYZ1M3 can improve gut microbiota dysbiosis in UC mice, increase microbial richness, reduce harmful bacteria, and elevate butyrate levels in cecal contents, suggesting that it may exert anti-inflammatory and barrier-protective effects by improving SCFA metabolism (70).

The combination of *Bifidobacterium longum* Bif10 and Bif11 with maltose has been shown to increase cecal butyrate levels, promote IL-10 and IL-22 secretion, and induce the expression of antimicrobial peptides such as RegIIIβ and S100A8, thereby enhancing mucosal defense (88, 89) (**Table 1**). Research on sulfated polysaccharides from *Laminaria japonica* further indicates that natural polysaccharides can act as prebiotic-like substrates, alleviating colitis by remodeling the gut microbiota and promoting butyrate-mediated barrier repair (90). This supports the “probiotics/prebiotics-SCFAs-barrier repair” axis as an important mechanism in microbiota-based interventions for UC.

In addition to SCFAs, tryptophan metabolites are involved in the regulation of mucosal immune homeostasis. *A. muciniphila* and its outer membrane protein Amuc_1100 can restore fecal microbiota-mediated tryptophan metabolic enrichment, modulate levels of kynurenine (Kyn) (91), picolinic acid (PIC), and indole-3-acetic acid (IAA) (92), and upregulate aryl hydrocarbon receptor (AhR) target genes, including CYP1A1, IL-10, and IL-22 (91). These effects may reduce colonic inflammation and help preserve barrier integrity. Studies on *Limosilactobacillus reuteri* also show that it can reverse abnormal intestinal metabolic profiles, with differential metabolites mainly enriched in linoleic acid metabolism (61), tryptophan metabolism, and unsaturated fatty acid biosynthesis (93), suggesting that lipid and amino acid metabolic remodeling may provide additional mechanisms for probiotic-mediated mucosal barrier repair.

### 4.5 Modulating immune responses and NF-κB/AhR/NLRP3 crosstalk

Probiotics and postbiotics may modulate epithelial and immune-cell responses through pattern-recognition receptors, cytokine networks, and intracellular inflammatory signaling pathways (94). Commonly reported effects include reductions in TNF-α, IL-1β, IL-6, and IL-17, as well as increases in IL-10, TGF-β, IL-22, and secretory immunoglobulin A (SIgA) (95). SIgA contributes to immune exclusion by limiting pathogen binding to epithelial receptors and reducing microbial penetration of the mucus layer.

The NF-κB, AhR, and NLRP3 pathways form an interconnected regulatory network involved in mucosal inflammation and barrier injury (96). NF-κB is a central transcriptional regulator of inflammatory cytokines and can be activated by pathogen-associated molecular patterns, epithelial injury, oxidative stress, and pro-inflammatory cytokines (97). Probiotic strains or their metabolites may suppress excessive NF-κB activation, thereby reducing TNF-α, IL-1β, IL-6, and chemokine production.

AhR is activated by tryptophan-derived indole metabolites and contributes to epithelial homeostasis, mucus production, IL-22-mediated repair, and immune tolerance (98). AhR activation may also restrain excessive NF-κB signaling, whereas impaired tryptophan metabolism and reduced AhR ligand availability may exacerbate inflammatory responses (99). In parallel, NLRP3 inflammasome activation requires priming signals, often involving NF-κB, followed by activation signals such as reactive oxygen species, mitochondrial dysfunction, extracellular ATP, or microbiota-derived danger signals (100). Activated NLRP3 promotes caspase-1-dependent maturation of IL-1β and IL-18, which can amplify mucosal inflammation and barrier disruption (101).

Accordingly, probiotics may protect the intestinal barrier through coordinated regulation of this signaling network: suppression of NF-κB-dependent inflammatory priming, enhancement of AhR-mediated epithelial and immune homeostasis, reduction of oxidative stress, and attenuation of aberrant NLRP3 inflammasome activation. *Parabacteroides distasonis*, for example, has been reported to exert anti-inflammatory, immune-regulatory, microbiota-modulating, and barrier-repair effects, potentially including regulation of the Th17/Treg balance (102). However, the specific pathways involved vary substantially among strains and experimental settings.

### 4.6 Attenuating oxidative stress and promoting epithelial repair

Oxidative stress is an important mechanism underlying intestinal mucosal injury in UC (103). Under inflammatory conditions, increased infiltration of neutrophils and macrophages leads to excessive production of reactive oxygen species (ROS) and reactive nitrogen species (RNS) (104), causing lipid peroxidation, DNA damage, mitochondrial dysfunction, and intestinal epithelial cell apoptosis. These processes further promote tight junction protein degradation, mucus layer disruption, and increased intestinal permeability (105).

Studies have reported that opportunistic bacterial infection can recruit neutrophils by inducing IL-8 release (106); neutrophils then release ROS and proteases (107), further damaging epithelial junctions. Meanwhile, M1 macrophages secrete TNF-α and IL-1β, which can suppress tight junction protein expression and aggravate barrier disruption (108). Probiotics may reduce oxidative stress and promote epithelial repair through above several mechanisms. On the one hand, *Bifidobacterium*, *Lactobacillus*, and related genera can remodel the gut microbiota, increase metabolites such as SCFAs and indole derivatives, and activate host antioxidant defense systems, possibly involving the Nrf2/HO-1 pathway (109). They can also inhibit NF-κB and NLRP3 inflammasome activation, thereby reducing pro-inflammatory cytokine release and inflammatory cell infiltration (110).

On the other hand, probiotic-derived SCFAs can activate MAPK/ERK signaling, upregulate ZO-1, Occludin, and Claudin-1 expression, promote the localization of junctional complexes to the cell membrane, and restore mechanical barrier integrity. In addition, probiotics can promote IL-10 and IL-22 secretion, enhance the expression of antimicrobial peptides such as RegIIIβ and S100A8, and improve the mucus layer and chemical barrier (88, 89). Tryptophan metabolites, such as indole-3-propionic acid, may regulate the TLR4/NF-κB, Wnt/β-catenin pathway, thereby promoting epithelial cell proliferation, migration, and mucosal healing (111, 112).

Recent research on “prebiotic-augmented nanozyme-armored probiotics” further suggests that constructing a nano-protective layer with antioxidant enzyme-like activity on the surface of probiotics, together with prebiotics to enhance probiotic colonization and metabolic activity, can simultaneously achieve ROS scavenging, microbiota remodeling, and barrier repair in the inflamed intestinal microenvironment (113). This approach offers a new strategy for intervention in UC and intestinal inflammation-related systemic disorders. Thus, antioxidant defense and epithelial repair are important complementary mechanisms by which probiotics protect the intestinal mucosal barrier in UC.

### 4.7 Strain specificity, safety considerations, and multi-strain probiotic formulations

The effects of probiotics should not be generalized at the genus or species level (114). Their efficacy depends on the specific strain, dose, formulation, viability, treatment duration, baseline microbiota composition, disease activity, mucosal permeability, host immune status, diet, and concomitant medications. A strain that is beneficial in one experimental model or patient subgroup may be ineffective, or potentially unfavorable, in another context.

Under conditions of severe dysbiosis, marked barrier disruption, immunosuppression, or critical illness, live microbial preparations may fail to confer benefit and may theoretically increase bacterial translocation, inflammatory activity, bacteremia, or infection risk in susceptible individuals (115). Moreover, mucus-degrading bacteria, highly immunostimulatory strains, or products with inadequate quality control may have unfavorable effects under specific ecological conditions. Therefore, therapeutic conclusions should be based on strain-specific evidence, standardized product characterization, and well-designed clinical trials rather than broad assumptions regarding “probiotics” as a uniform category.

Multi-strain probiotic formulations may offer functional complementarity by combining strains with different capacities for pathogen inhibition, SCFA production, mucus-layer support, epithelial repair, antioxidant activity, and immune regulation. Such combinations may theoretically target multiple pathogenic processes in UC simultaneously. However, the superiority of multi-strain formulations over single-strain preparations has not been consistently established (116). Strain compatibility, dose ratios, colonization competition, metabolic interactions, product stability, and genuine synergistic effects remain insufficiently standardized (117). Considerable heterogeneity in strain composition, administration schedules, disease severity, and outcome measures currently limits cross-study comparison and clinical extrapolation.

In summary, probiotics, postbiotics, and probiotic-derived EVs may protect the intestinal mucosal barrier through coordinated modulation of microbial ecology, metabolites, epithelial tight junctions, mucus secretion, oxidative stress, and immune-inflammatory pathways. Nevertheless, their efficacy and safety are strain-specific and context-dependent. Future studies should prioritize standardized strain identification, product quality control, mechanistic validation of postbiotics and EVs, and well-designed clinical trials evaluating single-strain and multi-strain interventions in defined UC populations.

## 5 Research progress on major probiotics in UC treatment

### 5.1 Lactobacillus

*Lactobacillus* is one of the most frequently studied probiotic genera in UC research (118). It exerts barrier-protective effects by producing lactic acid, inhibiting pathogens, strengthening tight junctions, promoting mucin expression, and regulating inflammatory cytokines. *L. reuteri* ZY15 can improve intestinal barrier function by upregulating Muc1, Muc2, Occludin, ZO-1, and Claudin-1 expression, while reducing IL-1β and TNF-α levels (25) (**Table 1**). In a 5-FU-induced intestinal mucosal injury model, *Limosilactobacillus reuteri* attenuated body weight loss, colon shortening, and histopathological injury, and increased the expression of ZO-1, Occludin, Claudin-4, Muc1, and Muc2, providing experimental evidence for its application in mucosal injury-related diseases (119).

### 5.2 Bifidobacterium

*Bifidobacterium* is a beneficial genus involved in maintaining intestinal microecological stability (120). *B. infantis* B8762 can downregulate pro-inflammatory cytokines, alleviate colitis symptoms, and protect colonic structure (121). *B. infantis* FJSYZ1M3 can increase microbial species richness in UC mice, reduce the abundance of harmful bacteria, and elevate butyrate levels in cecal contents (70). In addition, the combination of *B. infantis* ATCC 15697 with xylo-oligosaccharides can enhance colonic epithelial barrier integrity (122), suggesting that combined probiotic and prebiotic administration may have synergistic effects (**Table 1**).

### 5.3 Akkermansia muciniphila

*Akkermansia muciniphila* is a next-generation probiotic that has attracted increasing attention in recent years. Its abundance is often reduced in patients with UC (123), suggesting that its depletion may be associated with mucosal barrier disruption and aggravated inflammation. Animal studies have shown that supplementation with *A. muciniphila* can alleviate DSS-induced mucosal inflammation, reduce inflammatory cytokine levels, and enhance barrier function (124). These effects may be associated with increased IL-10 release, regulation of tryptophan metabolism, activation of AhR signaling, and upregulation of IL-22 expression (125) (**Table 1**).

### 5.4 Parabacteroides

*Parabacteroides distasonis* is a member of the core human gut microbiota and has anti-inflammatory, immunomodulatory, and barrier-repairing properties (126, 127). Studies have shown that *P. distasonis* can improve body weight loss (128, 129), colon shortening, bleeding, and abnormal stool consistency in UC model mice, while reducing intestinal mucosal injury and restoring barrier function. Its capacity to regulate the Th17/Treg balance also provides a promising direction for immune intervention in UC.

## 6 Prospects for combined treatment of UC with probiotics, prebiotics, active components of traditional Chinese medicine, and novel delivery systems

Although probiotics may alleviate UC by modulating gut microbiota composition, promoting SCFA production, suppressing inflammatory responses, and repairing the intestinal mucosal barrier, UC is a heterogeneous disease involving microbial dysbiosis, epithelial injury, immune dysregulation, oxidative stress, and altered intestinal metabolism. Therefore, intervention with a single probiotic strain or a single therapeutic mechanism may be insufficient for many patients. Combined approaches involving probiotics, prebiotics, active components of traditional Chinese medicine (TCM), postbiotics, FMT, and advanced delivery systems have consequently become important directions for microbiota-based UC therapy.

### 6.1 Probiotics and prebiotics

Prebiotics are selectively utilized substrates that can promote the growth or metabolic activity of beneficial microorganisms. When administered together with probiotics, they form synbiotics, which may improve probiotic survival, colonization, metabolic activity, and functional persistence in the gastrointestinal tract (130, 131). Common prebiotics, including inulin, fructo-oligosaccharides, galacto-oligosaccharides, resistant starch, and lactulose, may promote the proliferation of beneficial taxa such as *Lactobacillus* and *Bifidobacterium*, lower colonic pH, increase SCFA production, and suppress the expansion of certain opportunistic pathogens (132).

The beneficial effects of synbiotics may extend beyond changes in bacterial abundance. Increased production of butyrate and other SCFAs may support epithelial energy metabolism, stimulate mucus secretion, enhance tight-junction protein expression, promote regulatory T-cell differentiation, and reduce excessive inflammatory signaling. In DSS-induced colitis models, synbiotic formulations containing *Lactobacillus*, *Bifidobacterium*, fructo-oligosaccharides, and inulin have been reported to reduce disease activity index (DAI), decrease the relative abundance of pathogenic bacteria, regulate immune-cell and epithelial-cell functions, and reduce serum IL-6 and IL-8 levels. However, the efficacy of synbiotics depends on the compatibility between the administered strains and substrate. A prebiotic that benefits one microbial community may have limited efficacy, or may cause excessive gas production and gastrointestinal discomfort, in another host setting. Therefore, rational synbiotic design should consider strain-specific substrate utilization, baseline microbiota composition, disease activity, and dietary intake.

### 6.2 Synergistic mechanisms of TCM herbal formulas, active components, and probiotics

Active components of TCM may act synergistically with probiotics because they can simultaneously influence the intestinal microbial ecosystem, inflammatory microenvironment, epithelial barrier, and host metabolism (133). TCM-derived polysaccharides, polyphenols, flavonoids, alkaloids, and organic acids may provide fermentable substrates for commensal microorganisms, selectively enrich beneficial bacterial populations, and modify microbial metabolic networks. For example, Scutellaria baicalensis polysaccharide SP2-1, sea buckthorn polysaccharides, and bergamot polysaccharides have been reported to promote the growth of beneficial bacteria, including *Bifidobacterium*, *Lactobacillus*, and *Akkermansia*, while increasing SCFA production (134).

The potential synergy between TCM components and probiotics may occur through several interconnected mechanisms. First, TCM-derived polysaccharides and other fermentable compounds may improve nutrient availability for beneficial microorganisms and increase the production of SCFAs, indole derivatives, and other microbial metabolites (135). Second, anti-inflammatory and antioxidant TCM constituents may reduce oxidative stress, suppress inflammatory cytokine release, improve mucus secretion, and enhance the expression of tight-junction proteins (136). These changes may create a more favorable intestinal environment for probiotic survival, colonization, and function. Third, probiotics may participate in the biotransformation of TCM constituents, converting poorly absorbed parent compounds into metabolites with altered bioavailability, stability, or biological activity (137).

This bidirectional interaction may involve SCFA-related signaling, the tryptophan-AhR axis, NF-κB/NLRP3 inflammatory pathways, and mucosal immune homeostasis. For example, increased SCFA production may promote epithelial repair and anti-inflammatory immune responses, whereas tryptophan-derived microbial metabolites may activate AhR and support IL-22-associated barrier protection. At the same time, TCM-derived antioxidant and anti-inflammatory compounds may suppress NF-κB activation and reduce NLRP3 inflammasome-associated IL-1β and IL-18 release. Traditional formulas, including Yiyi Fuzi Baijiang Powder (138), Shenling Baizhu Powder (139), and Banxia Xiexin Decoction (140), have been reported to regulate intestinal microecology and alleviate experimental UC through mechanisms involving NF-κB signaling, microbiota-amino acid metabolism, and microbiota-metabolite-inflammation interactions.

Natural compounds such as ferulic acid may further improve the inflammatory intestinal microenvironment through antioxidant, anti-inflammatory, and barrier-protective effects, thereby supporting probiotic activity (141). Nevertheless, current evidence for TCM-probiotic synergy is derived predominantly from animal experiments, *in vitro* studies, and metabolomic associations. The active constituents responsible for efficacy, the key microbial strains involved in biotransformation, and the causal relationships between microbial metabolites and clinical benefit remain insufficiently defined. Future studies should combine chemical characterization, strain-resolved metagenomics, targeted metabolomics, and controlled clinical trials to establish reproducible TCM-probiotic combinations.

### 6.3 Postbiotics as complementary or alternative interventions

Postbiotics, including inactivated microbial cells, cell-wall components, extracellular polysaccharides, cell-free supernatants, microbial enzymes, and microbial metabolites, may provide an alternative or complement to live probiotic preparations (142). Compared with viable microorganisms, postbiotics may offer improved product stability, easier dose standardization, reduced storage requirements, and potentially lower risks of microbial translocation or infection in vulnerable patients.

Postbiotic components may preserve several beneficial functions of probiotics, including regulation of tight-junction proteins, attenuation of oxidative stress, modulation of NF-κB/NLRP3 signaling, enhancement of mucus secretion, and regulation of immune-cell activity (143). SCFAs, indole derivatives, EPS, peptidoglycan fragments, and probiotic-derived extracellular vesicles may all participate in epithelial and immune signaling (144). However, postbiotics should not be regarded as universally interchangeable with live probiotics because their biological activity depends on the producing strain, culture conditions, processing method, component composition, dose, and target population (145). More standardized definitions and rigorous clinical studies are needed before postbiotics can be broadly integrated into UC management.

### 6.4 Sequential FMT-probiotic therapy

FMT can reconstruct a disrupted microbial community more comprehensively than conventional probiotic supplementation because it transfers a complex microbial ecosystem and associated functional capacity from a healthy donor to a recipient (146). In UC, FMT may rapidly increase microbial diversity, restore depleted commensal taxa, improve SCFA-related metabolic functions, and reduce inflammation-associated microbial signatures (147, 148). However, the long-term stability of the transplanted microbial community remains variable, and some recipients may experience partial loss of donor-derived functions or disease relapse after the initial response.

Sequential FMT-probiotic therapy has therefore been proposed as a potential strategy to improve post-transplant microbiota stability. In this model, FMT is first used to reconstruct the disrupted microbial ecosystem and functional network, followed by administration of selected probiotics or synbiotics to support beneficial microbial functions, reinforce barrier repair, and potentially reduce relapse risk (149). Probiotics administered after FMT may help maintain SCFA production, suppress opportunistic pathogen expansion, enhance colonization resistance, and support epithelial and immune homeostasis. Synbiotics may further provide substrates that facilitate the persistence or metabolic activity of beneficial microbial populations after transplantation (150).

Nevertheless, the clinical performance of sequential FMT-probiotic therapy may be influenced by donor selection, donor screening procedures, stool processing methods, FMT route, administration frequency, bowel preparation, antibiotic exposure, timing of post-FMT probiotic administration, probiotic strain selection (151), treatment duration, and concomitant medications. Baseline microbiota composition, mucosal inflammation, diet, and host immune status may also determine whether the administered probiotics can persist or function after FMT. At present, evidence supporting this sequential strategy remains limited. Standardized randomized controlled trials with long-term follow-up are required to determine the optimal treatment sequence, interval, probiotic formulation, maintenance duration, and safety profile.

### 6.5 Novel delivery systems for precision probiotic therapy

Novel delivery technologies may improve the precision and therapeutic efficiency of probiotics, postbiotics, and microbial metabolites. Smart hydrogels (152), microcapsules (153), nanocarriers (154), pH-responsive materials, and nanozyme shells (113) can protect live microorganisms from gastric acid, bile salts, digestive enzymes, and the oxidative inflammatory environment. These systems may also improve colon-specific release, prolong intestinal retention, and enhance the local delivery of microbial metabolites or anti-inflammatory compounds.

Recent studies of prebiotic-augmented nanozyme-armored probiotics suggest that combining a prebiotic substrate, antioxidant nanozyme protection, and probiotics may simultaneously improve bacterial survival, scavenge excess ROS, reshape the microbiota, and promote barrier repair in inflamed intestinal tissue (113). Such integrated systems may be particularly valuable in UC, where oxidative stress, impaired mucus integrity, and altered luminal conditions can limit probiotic viability and function. However, the long-term biosafety, biodegradability, manufacturing reproducibility, regulatory classification, and cost-effectiveness of these delivery platforms require careful evaluation before clinical translation (**Figure 2**).

**Figure 2:**
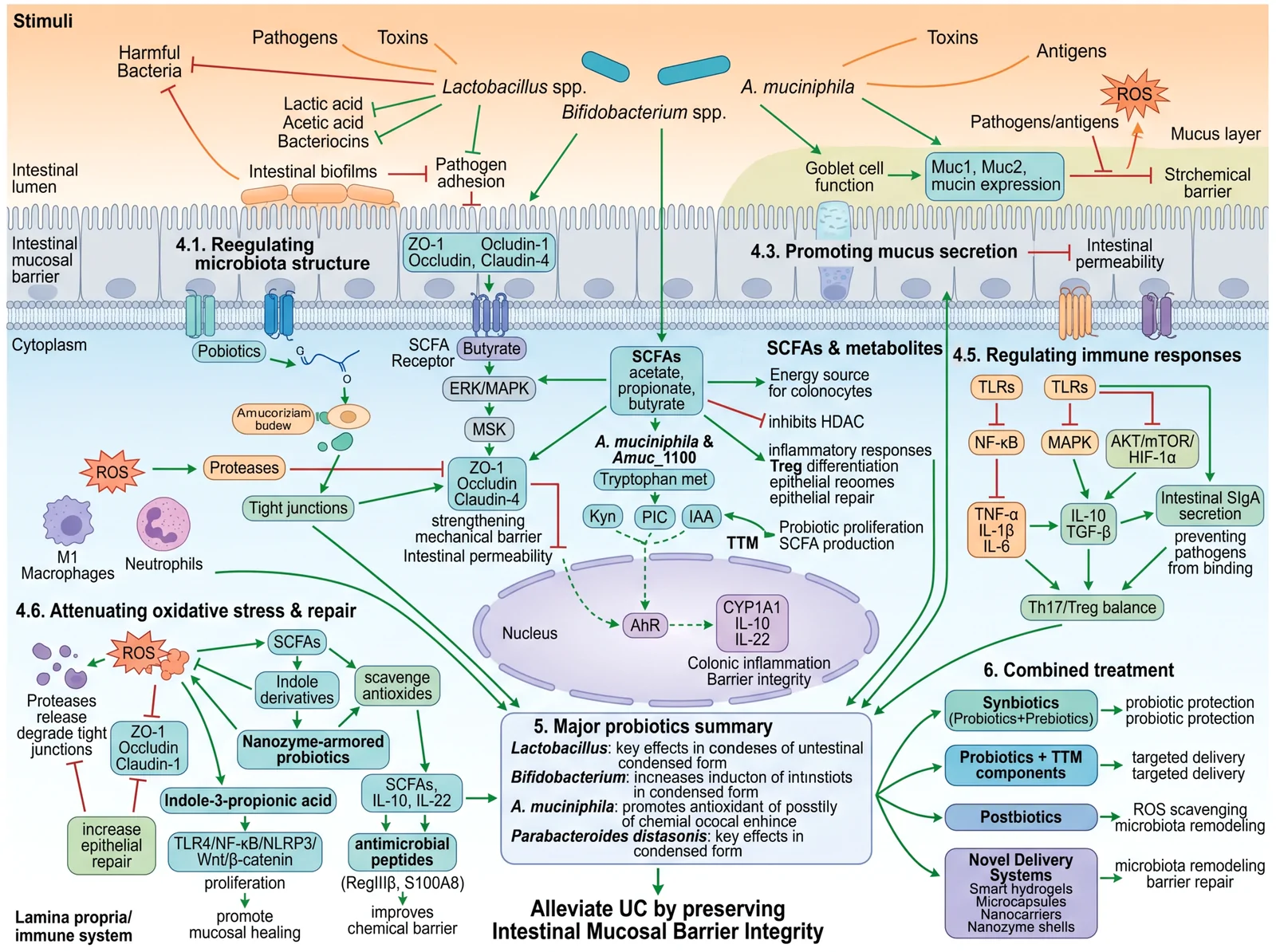
Probiotic-mediated protection of the intestinal mucosal barrier in ulcerative colitis. Schematic illustration of the mechanisms through which probiotics protect intestinal mucosal barrier integrity and alleviate ulcerative colitis (UC). The figure summarizes six interconnected modules: regulation of gut microbiota composition, reinforcement of the epithelial mechanical barrier, promotion of mucus secretion, regulation of mucosal immune responses, attenuation of oxidative stress and promotion of epithelial repair, and combined therapeutic strategies. Probiotic strains and probiotic-associated microorganisms, including *Lactobacillus* spp., *Bifidobacterium* spp., *Akkermansia muciniphila*, and *Parabacteroides distasonis*, may improve microbial homeostasis by inhibiting pathogen adhesion, reducing biofilm formation, producing antimicrobial substances, and enhancing colonization resistance. They may also strengthen the epithelial barrier by increasing the expression or integrity of tight-junction proteins, including ZO-1, occludin, claudin-1, and claudin-4, thereby reducing intestinal permeability. In parallel, probiotics may promote goblet-cell function, mucus secretion, and mucin expression, particularly MUC1 and MUC2, and thus support restoration of the mucus barrier. Probiotic-derived metabolites, especially short-chain fatty acids (SCFAs), butyrate, and tryptophan-derived metabolites, may act through SCFA receptors and signaling pathways involving ERK/MAPK/MSK and the aryl hydrocarbon receptor (AhR). These pathways contribute to epithelial energy metabolism, tight-junction maintenance, immune regulation, and barrier homeostasis. Probiotics may further reduce mucosal injury by limiting reactive oxygen species (ROS) accumulation, enhancing antioxidant activity, suppressing inflammatory signaling through NF-κB, MAPK, AKT/mTOR/HIF-1α, and NLRP3 pathways, and promoting epithelial repair. The lower-right module summarizes potential combination strategies. Synbiotics, probiotic formulations combined with traditional Chinese medicine-derived components, postbiotics, and novel delivery systems may improve probiotic stability and survival, facilitate colon-targeted delivery, promote microbiota remodeling, enhance ROS scavenging, and support mucosal barrier repair. These approaches may therefore serve as adjunctive strategies for UC management. The arrows indicate proposed regulatory or functional relationships, whereas inhibitory lines indicate suppression of the corresponding process. The figure is schematic and does not imply that every strain or intervention activates all pathways shown.

## 7 Limitations and safety issues

Despite their potential to prevent and manage UC, the clinical use of probiotics remains constrained by several challenges. First, probiotic effects are highly strain-specific. Different strains vary in adhesion capacity, acid-producing ability, immunomodulatory effects, metabolite profiles, and safety; therefore, therapeutic efficacy cannot be inferred simply from the genus or species. Second, host-related factors vary substantially. Age, disease activity, dietary patterns, baseline microbiota composition, immune status, and concomitant medications may all influence the response to probiotic intervention. Third, further standardization is needed for probiotic preparations, including viable bacterial counts, stability, acid and bile salt tolerance, storage conditions, and quality control criteria.

Moreover, probiotics are not beneficial under all circumstances. Some studies suggest that, in certain host conditions or when the gut microbiota is not yet mature, supplementation with specific probiotics may fail to produce the expected effects and may even aggravate symptoms such as diarrhea. Therefore, clinical use of probiotics should take into account indication selection, strain source, dosage and treatment duration, safety monitoring, and efficacy assessment. Greater caution is warranted in patients with severe UC, marked immunosuppression, or a risk of bacteremia.

At present, evidence for probiotic treatment of UC is still mainly derived from animal experiments, *in vitro* studies, and some small-scale clinical trials. Considerable differences among studies in strain type, dose, treatment duration, route of administration, disease model, and outcome measures limit the comparability and reproducibility of the findings.

## 8 Perspectives

With advances in metagenomics, metatranscriptomics, metabolomics, proteomics, and single-cell sequencing, research on probiotic therapy for UC is moving from empirical supplementation toward more precise, mechanism-based, and individualized intervention. Future studies may focus on several areas.

First, specific strains with clearly defined barrier-protective and immunomodulatory effects should be screened, and standardized systems for functional evaluation should be established. Second, multi-omics approaches should be used to clarify the key mechanisms by which probiotics regulate tight junctions, the mucus barrier, metabolic pathways, and immune cell differentiation. Third, it is necessary to define patterns of response to probiotic therapy in patients at different disease stages, with different inflammatory phenotypes, and with distinct baseline microbiota profiles. Fourth, combined intervention models involving probiotics, prebiotics, postbiotics, active components of traditional Chinese medicine, biologics, and novel delivery systems should be further explored. Fifth, quality control, safety evaluation, and clinical translation of probiotic preparations should be strengthened.

Precision probiotic therapy may become an important component of integrated UC management. Individualized intervention strategies based on the patient’s microbiota structure, metabolic profile, and immune status may help improve therapeutic efficacy, reduce relapse risk, and limit adverse drug reactions.

## 9 Conclusion

Intestinal mucosal barrier injury and gut microbiota dysbiosis are central events in the onset and progression of UC. Probiotics can protect the intestinal mucosal barrier and alleviate intestinal inflammation by remodeling the gut microbiota, enhancing colonization resistance, upregulating tight junction protein expression, promoting mucus and mucin secretion, regulating microbial metabolites such as SCFAs and tryptophan derivatives, inhibiting pro-inflammatory signaling pathways, promoting SIgA secretion, and maintaining immune balance, including the Th17/Treg axis.

*Lactobacillus*, *Bifidobacterium*, *Akkermansia muciniphila*, and *Parabacteroides distasonis* have all shown therapeutic potential in UC-related studies. However, probiotic efficacy is influenced by multiple factors, including strain specificity, host status, formulation quality, and study design. Further validation through standardized basic research and high-quality clinical trials is still required. Overall, interventions targeting intestinal probiotics and their metabolites may become an important adjunctive strategy for UC treatment and maintenance of remission.
